# Comparative Antihyperglycemic and Antihyperlipidemic Effects of Lawsone Methyl Ether and Lawsone in Nicotinamide-Streptozotocin-Induced Diabetic Rats

**DOI:** 10.3390/metabo13070863

**Published:** 2023-07-20

**Authors:** Muhammad Khan, Muhammad Ajmal Shah, Mustafa Kamal, Mohammad Shamsul Ola, Mehboob Ali, Pharkphoom Panichayupakaranant

**Affiliations:** 1 Department of Pharmacognosy and Pharmaceutical Botany, Faculty of Pharmaceutical Sciences, Prince of Songkla University, Hat-Yai 90112, Thailand; 6210730010@email.psu.ac.th; 2 Department of Pharmacology, Federal Urdu University of Arts, Science and Technology, Karachi 75300, Pakistan; 3 Department of Pharmacy, Hazara University, Mansehra 21300, Pakistan; 4 Department of Zoology, Abdul Wali Khan University Mardan, Mardan 23200, Pakistan; mustafakamal@awkum.edu.pk; 5 Department of Biochemistry, College of Science, King Saud University, Riyadh 11451, Saudi Arabia; mola@ksu.edu.sa; 6 Senior Scientist Toxicology Invivotek Nexus, a Genesis Biotech Group LLC Company, 17 Black Forest RD, Hamilton, NJ 08690, USA; mali@invivotek.com; 7 Phytomedicine and Pharmaceutical Biotechnology Excellence Center, Faculty of Pharmaceutical Sciences, Prince of Songkla University, Hat-Yai 90112, Thailand

**Keywords:** diabetes mellitus, hyperglycemic, hyperlipidemia, lawsone, lawsone methyl ether

## Abstract

Our previous study uncovered potent inhibitory effects of two naphthoquinones from *Impatiens balsamina*, namely lawsone methyl ether (2-methoxy-1,4-naphthoquinone, LME) and lawsone (2-hydroxy-1,4-naphthoquinone), against α-glucosidase. This gave us the insight to compare the hypoglycemic and hypolipidemic effects of LME and lawsone in high-fat/high-fructose-diet- and nicotinamide-streptozotocin-induced diabetic rats for 28 days. LME and lawsone at the doses of 15, 30, and 45 mg/kg, respectively, produced a substantial and dose-dependent reduction in the levels of fasting blood glucose (FBG), HbA1c, and food/water intake while boosting the insulin levels and body weights of diabetic rats. Additionally, the levels of total cholesterol (TC), triglycerides (TGs), high-density lipoproteins (HDLs), low-density lipoproteins (LDLs), aspartate transaminase (AST), alanine transaminase (ALT), creatinine, and blood urea nitrogen (BUN) in diabetic rats were significantly normalized by LME and lawsone, without affecting the normal rats. LME at a dose of 45 mg/kg exhibited the most potent antihyperglycemic and antihyperlipidemic effects, which were significantly comparable to glibenclamide but higher than those of lawsone. Furthermore, the toxicity evaluation indicated that both naphthoquinones were entirely safe for use in rodent models at doses ≤ 50 mg/kg. Therefore, the remarkable antihyperglycemic and antihyperlipidemic potentials of LME make it a promising option for future drug development.

## 1. Introduction

Diabetes mellitus (DM) is a chronic metabolic disorder characterized by hyperglycemia and disturbances of carbohydrate, fat, and protein metabolism resulting from defects in insulin secretion, insulin action, or both. Insulin is a hormone produced by the pancreas, and it plays a crucial role in regulating the uptake and utilization of glucose in the body. In type 1 diabetes, the pancreas does not produce enough insulin, while in type 2 diabetes, the body is unable to effectively utilize the insulin it produces [1]. DM is indeed the most prevalent metabolic disease of the contemporary age. Therefore, it is termed a “modern-day epidemic”. It is a growing global public health issue that compromises living standards and places an undue financial burden on the healthcare systems. Based on the International Diabetes Federation (IDF), DM has affected around 463 million individuals in 2019. Of these, 4.2 million patients succumbed to death, costing a total of USD 760 billion in diabetes-related medical expenses [2].

Aside from glucose metabolism, insulin also regulates lipid metabolism. Hence, diabetic patients with poorly controlled glycemic levels frequently develop dyslipidemias. This implies that any fluctuations in insulin level or action may disrupt the body’s lipid profiles, which usually leads to cardiovascular diseases [3]. Although controlling glycemic levels is a fundamental approach to managing DM, normalizing elevated lipid levels in diabetic individuals should also be a therapeutic necessity. For this purpose, a plethora of conventional antihyperglycemic and antihyperlipidemic medications are commercially available.

Insulin analogs, biguanides, sulfonylureas, thiazolidinedione (TZD), dipeptidyl peptidase-4 (DPP-4) inhibitors, sodium-glucose cotransporter (SGLT-2) inhibitors, and glucagon-like peptide-1 (GLP-1) receptor agonists are the major classes of antidiabetic medications [4]. On the other hand, statins, fibric acid derivatives, nicotinic acid derivatives, bile acid binding resins, and cholesterol absorption inhibitors are the commonly used antihyperlipidemic agents [5,6]. Although these conventional antihyperglycemic and antihyperlipidemic drugs are commonly prescribed, they are expensive, less efficacious, produce tolerance, and have undesirable side effects [7,8,9]. Biguanides (metformin) can cause anemia and neuropathy. DPP-4 inhibitors (sitagliptin) and sulfonylureas (glipizide) have the potential to cause hypoglycemia, weight gain, nausea, vomiting, headache, and dizziness. Similarly, insulin analogs and GLP-1 agonists (liraglutide), apart from being expensive, can cause hypoglycemia, allergy, and injection site reactions [10]. Statins, which are the most commonly used antihyperlipidemic drugs, can cause severe side effects like myopathy, rhabdomyolysis, nephrotoxicity, cardiomyopathy, and elevated serum transaminases. Recent clinical trials have linked statin use with an increase in the incidence of type 2 DM [11]. Ezetimibe is also associated with unwanted effects in the form of headaches, abdominal pain, and diarrhea. Moreover, it elevates the functional markers of the liver, namely alanine transaminase and aspartate transaminase [12].

The aforementioned limitations associated with conventional antihyperglycemic and antihyperlipidemic medications emphasize the necessity for novel drugs that offer improved efficacy and safety in lowering blood sugar and lipid levels. The World Health Organization (WHO) has also recommended the use of traditional plants for the treatment of DM, due to their effectiveness, non-toxic nature, and minimal or no side effects. These plant-based treatments are considered excellent options for oral therapy in the management of DM [13]. This research impetus led to the discovery of phytochemicals with remarkable antihyperglycemic potentials including berberine, curcumin, resveratrol, quercetin, epigallocatechin gallate (EGCG), etc. [14]. Plumbagin, shikonin, and rhinacanthin-C are noteworthy among these phytochemicals. They are the naphthoquinones, which not only demonstrate marvelous antihyperglycemic effects but also exhibit promising antihyperlipidemic activities [15,16,17]. Likewise, lawsone methyl ether (LME) and lawsone are naphthoquinones found in *Impatiens balsamina* L. [18]. Our previous in silico and in vitro studies uncovered potent inhibitory actions of LME and lawsone against α-glucosidase with excellent in silico ADMET (absorption, distribution, metabolism, excretion, and toxicity) profiles, revealing no toxicity in terms of tumorigenic, irritant, and reproductive effects [19]. This increased our interest in validating the in silico and in vitro results using animal models. Therefore, the present study aimed to investigate the antihyperglycemic and antihyperlipidemic effects of LME and lawsone in high-fat/high-fructose diet (HFFD)- and nicotinamide-streptozotocin (NA-STZ)-induced diabetic rats. Currently, LME can be semi-synthesized from lawsone with a high yield and low cost of production [20]. Thus, it might be a promising option for future drug development. Notably, this is a comprehensive and comparative study to discover new antidiabetic candidates that possess hypoglycemic and hypolipidemic potentials for further drug development.

## 2. Materials and Methods

### 2.1. Drugs and Chemicals

Lawsone, glibenclamide, streptozotocin (STZ), nicotinamide, cholesterol, and fructose were obtained from Sigma-Aldrich Chemie GmbH, Steinheim, Germany. LME was semi-synthesized by methylating lawsone using a method previously reported [20]. All the other chemicals and reagents were preferably of analytical grade.

### 2.2. Experimental Animals

A total of 90 adult male Wistar rats (6 weeks old), weighing approximately 130 ± 10 g, were obtained from the Pakistan Council of Scientific & Industrial Research (PCSIR) Laboratories Complex, Karachi. Before experimentation, the animals were allowed to acclimatize in the laboratory for almost one week and fed on a normal chow diet. The animals were provided with free access to food and water under standard environmental conditions at a room temperature of 24 ± 2 °C, humidity of 55 ± 10%, and 12 h/12 h of light/darkness. All experimental protocols were approved by the Institutional Animal Care and Use Committee, PCSIR Laboratories Complex, Karachi, Pakistan (Ref. PCSIR–KLC/IEC/2022/02).

### 2.3. Induction of Obesity, Hyperlipidemia, and Insulin Resistance

After acclimatization, the animals were randomly split into two groups. Their body weights along with various biochemical indices, including fasting blood glucose (FBG), total cholesterol (TC), triglyceride (TG), high-density lipoprotein (HDL), low-density lipoprotein (LDL), and insulin levels were determined before the commencement of diet interventions. One group was fed a normal chow diet, while another group was fed a high-fat/high-fructose diet (HFFD). This diet was formulated indigenously in our laboratory in accordance with the method described earlier [21], with a few modifications. HFFD contained high-calorie meals by adding Banaspati ghee (hydrogenated oil), coconut oil, and raw cholesterol to the normal chow diet. It also included an additional 30% of refined coarse fructose in the drinking water ad libitum. The given diet plans were followed for each group of rats for 10 weeks (and continued during the treatment period). At the end of the 10th week, blood samples were collected from the tail veins of both groups. Their body weights along with the relevant biochemical indices were measured to evaluate the induction of obesity, hyperlipidemia, and insulin resistance.

### 2.4. Standardization of STZ Dose for the Induction of DM

After the 10th week of dietary intervention, the rats manifesting obesity and dyslipidemia were fasting overnight. Subsequently, a pilot study was conducted using various concentrations of STZ (35, 40, and 45 mg/kg) to determine its optimal dose for inducing diabetes. Based on the results, a single intraperitoneal dose of STZ (40 mg/kg) diluted in 0.1 M cold citrate buffer (pH 4.5) with a pre-nicotinamide (100 mg/kg) injection to minimize pancreatic damage was standardized to induce DM [22]. After 72 h of NA-STZ injection, the animal’s tail vein was pricked to collect blood for the FBG determination using a glucometer (Accu-Chek, Roche, Burgdorf, Switzerland). Animals with an FBG level above 400 mg/dL and having symptoms of polyphagia, polydipsia, and polyuria were marked as diabetic.

According to the results of an acute toxicity investigation, the no observed adverse effect level (NOAEL) for LME and lawsone was 50 mg/kg. Furthermore, several previous studies reported varying doses of 1,4-napthoquinones that were therapeutically active in rodent models [15,16,17]. Based on these reports, three doses of LME and lawsone (15, 30, and 45 mg/kg) were selected for this experiment. In the present study, the antihyperglycemic and antihyperlipidemic effects of LME and lawsone were compared with the standard drug, glibenclamide. Its well-established mechanisms of action and widespread use in clinical practice make it a valuable drug for evaluating the antihyperglycemic effects of other compounds in rat models of diabetes. In addition, the ability of glibenclamide to reduce the elevated biomarkers of oxidative stress, liver function, kidney function, and lipid peroxidation during DM makes it a suitable choice to be used as a reference drug in antihyperglycemic and antihyperlipidemic assays [23,24].

Treatments with LME, lawsone, and glibenclamide (0.6 mg/kg) were started after 72 h of NA-STZ injection and were recorded as the first day. LME and lawsone were dissolved in a cosolvent system consisting of propylene glycol, Tween 80, and water (4:1:4). The required amounts of the drugs in a solution not exceeding 1 mL were administered once daily to the rats via feeding tubes for 28 days.

### 2.5. Experimental Design

The rats were experimentally assigned into 15 groups with six (6) rats per group as described below.

Group 1: normal control rats receiving cosolvent only.
Groups 2, 3, and 4: normal control rats receiving 15, 30, and 45 mg/kg of LME, respectively.
Groups 5,6, and 7: normal control rats receiving 15, 30, and 45 mg/kg of lawsone, respectively.
Group 8: diabetic control rats receiving cosolvent only.
Groups 9, 10, and 11: diabetic rats receiving 15, 30, and 45 mg/kg of LME, respectively.
Groups 12, 13, and 14: diabetic rats receiving 15, 30, and 45 mg/kg of lawsone, respectively.
Group 15: diabetic rats receiving 0.6 mg/kg of glibenclamide.

### 2.6. Determination of Body Weight, Food and Water Intake, and FBG

All animals’ initial and final body weights as well as their daily food and water consumption were recorded. The FBG was determined on days 0, 7, 14, 21, and 28 with the help of a glucometer using the blood drawn from the animals’ tails. On the final day of the experiment (the 28th day), the rats were fasted overnight and were euthanized with an intraperitoneal dose of phenobarbital ranging from 100 to 150 mg/kg. After performing a cardiac puncture, blood was collected from each rat and centrifuged at 4 °C and 800× *g* for 15 min. The collected serum was then utilized for various biochemical examinations.

### 2.7. Measurement of HbA1c and Insulin Levels

The iChroma™ HbA1c self-analyzer (Boditech Med Inc., Gangwon-do, Republic of Korea) was used to test HbA1c levels of whole blood samples. The electro-chemiluminescence technique with a Roche kit and a Cobas 6000 (Roche Diagnostics, Rotkreuz, Switzerland) was employed to detect insulin levels in serum.

### 2.8. Determination of Insulin Resistance and β-Cell Functioning Indices

Based on the fasting levels of insulin and blood glucose, the insulin resistance index (HOMA-IR) and β cell functioning index (HOMA-β) of all animals were calculated with the help of homoeostatic model assessment (HOMA) [25].

### 2.9. Measurement of Biochemical Indices for Lipid Profiles, Liver, and Kidney Functions

The levels of various biochemical indices, including total cholesterol (TC), triglycerides (TG), high-density lipoprotein (HDL), low-density lipoprotein (LDL), aspartate aminotransferase (AST), alanine aminotransferase (ALT), blood urea nitrogen (BUN), and creatinine were measured using standard kits (Martin Dow, Meymac, France) with the help of a hematology analyzer (Stat Fax 3300, Awareness Technology, FL, US). The following formulae were used to determine the atherogenic index (AI), atherogenic coefficient (AC), and cardiovascular risk index (CRI) [26].

AI=TC−HDL/HDL

AC=LDL/HDL

CRI=TC/HDL

### 2.10. Histopathological Findings of Pancreas

After blood sampling, the pancreases from euthanized rats were preserved in 10% neutral buffered formalin for 24 h. The specimens were washed in tap water, dehydrated using a graded alcohol series, cleared with xylene, and embedded in paraffin. Tissue blocks were prepared, and sections of 3–5 μm thickness were obtained using a microtome. The sections were mounted on glass slides, deparaffinized, and stained with hematoxylin-eosin (HE). Histopathological examination was performed at 10× magnification using a light microscope (Optika, Bergamo, Italy), and images were captured with a digital camera (Model B9, Optika, Bergamo, Italy) attached to the microscope at the microscopy unit of the Department of Pharmacy, COMSATS University Islamabad, Abbottabad Campus, Pakistan.

### 2.11. Statistical Analysis

The statistical analyses were performed using version 25 of the statistical software SPSS (SPSS Inc., Chicago, IL, USA). The analyses were conducted by using one-way ANOVA followed by Duncan’s multiple comparison test. The statistical significance was declared at *p* < 0.05. In accordance with Duncan’s multiple comparison test, all groups within the same parameter (e.g., food intake, ALT, or insulin levels) were compared not only with the control group but also with every other group within that parameter. The values obtained were expressed as means of six replicate determinations (*n* = 6) ± S.E.M.

## 3. Results and Discussion

### 3.1. Determination of a Non-Toxic Dose for LME and Lawsone

Prior to evaluating hypoglycemic and hypolipidemic activities, LME and lawsone were comprehensively evaluated for their possible hazardous effects using acute and sub-acute oral toxicity assays. Acute oral toxicity was conducted in mice according to the Organization of Economic Cooperation and Development (OECD) 423 guidelines [27]. In this study, animals were given a single oral dosage of LME (50, 300, and 2000 mg/kg) and lawsone (50 and 300 mg/kg) and monitored for 14 days to determine their acute toxic effects. In addition, a sub-acute toxicity study was carried out in Wistar rats by following the OECD 407 guidelines [28], with a few modifications. Based on previous reports, a 28-day repeated-dose oral toxicity study was performed using 15, 30, and 45 mg/kg of LME and lawsone [15,16,17]. The results of acute and sub-acute toxicity studies concluded that LME and lawsone were entirely safe for use in rodent models at doses ≤50 mg/kg.

### 3.2. Induction of Obesity, Hyperlipidemia, and Insulin Resistance

Obesity is a significant risk factor for type 2 DM. It contributes to the development of insulin resistance, which can progress to type 2 DM over the long run. High-calorie meals, particularly those rich in fats and carbohydrates, are the main contributors to obesity [29]. Using a high-fat (45% fat by energy) and high-fructose (30% fructose in drinking water ad libitum) diet in conjunction with NA-STZ injections was the focal point of the current study. This produced a perfect model of type 2 DM in adult male Wistar rats. This rat model precisely replicated the human stages of obesity, insulin resistance, prediabetes, and hyperlipidemias [30,31].

The effects of HFFD and a normal fat diet (NFD) on body weights, TC, TG, HDL, LDL, FBG, and insulin levels of rats fed for 10 weeks are illustrated in Table 1. Obesity was readily apparent in the rats fed an HFFD, which gained up to 45% more weight than the rats fed an NFD. Similarly, increased levels of TC, TG, and LDL but decreased levels of HDL were observed in HFFD-fed rats. However, no significant impact was found on the lipid profiles of rats given an NFD. In addition, FBG levels of rats given an HFFD were found to be considerably higher when compared to those rats fed a regular diet. In contrast, no marked difference was found in the insulin levels of the two groups. The overall results clearly indicated that rats fed an HFFD had developed obesity, hyperlipidemia, and insulin resistance, characteristics of prediabetes.

### 3.3. Effects of LME and Lawsone on Body Weights, Food, and Water Consumptions

Hyperglycemia, a typical characteristic of DM, frequently appears as a range of symptoms, such as polydipsia (unusual thirst), polyphagia (extreme hunger), polyuria (frequent urination), and unexplained weight loss [32]. Insulin deficiency primarily results in two major clinical outcomes: hyperglycemia and protein catabolism. Hyperglycemia manifests itself in the form of increased hunger, unusual thirst, and frequent urination. Conversely, protein catabolism leads to muscle atrophy and weight loss. These symptoms are the characteristics of the metabolic imbalances caused by insufficient insulin levels in the body [33]. In this study, diabetic rats exhibited the aforementioned symptoms of DM after receiving NA-STZ for 72 h. Diabetic control rats consumed more food and water and lost weight faster than normal rats (Table 2). Interestingly, body weight and water/food intake in diabetic rats were markedly normalized in a dose-dependent manner after oral administration of LME and lawsone, with no effect on normal rats. These findings suggest that LME and lawsone possess therapeutic benefits in managing weight and regulating water and food intake in diabetic conditions. Although glibenclamide (0.6 mg/kg) generated the most potent effects, they were statistically equivalent to the effects of LME and lawsone given at 45 mg/kg. The results indicated that hypoglycemic effects of LME and lawsone were demonstrated by their restoration of normal body weight, water intake, and food consumption in diabetic rats.

### 3.4. Effects of LME and Lawsone on FBG, HbA1c, and Insulin Levels

Insulin is a hormone of utmost importance in regulating the body’s metabolism. That is why any glitch in its action or production results in DM. In the current study, we firstly induced insulin resistance in rats by manipulating their diet. Subsequently, they received injections of NA-STZ, causing partial damage to the pancreatic ß-cells [34]. After 72 h of STZ injection, all of the rats showing elevated levels of FBG (above 400 mg/dL) and having symptoms of polyuria, polyphagia, polydipsia, and also weight loss were declared as diabetic.

**Figure 1:**
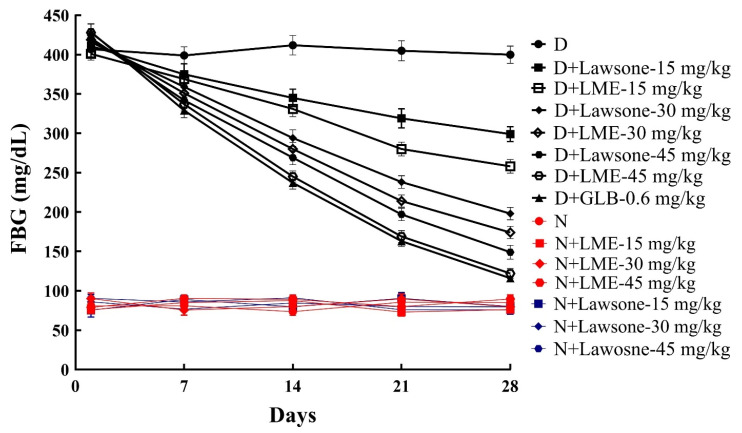
Comparative effects of LME and lawsone at weekly intervals on FBG levels. N: Nondiabetic control; D: Diabetic-control; N + LME or lawsone: nondiabetic receiving lawsone methyl ether or lawsone; D + LME or lawsone: Diabetic receiving lawsone methyl ether or lawsone; and D + GLB: Diabetic receiving glibenclamide.

**Figure 2:**
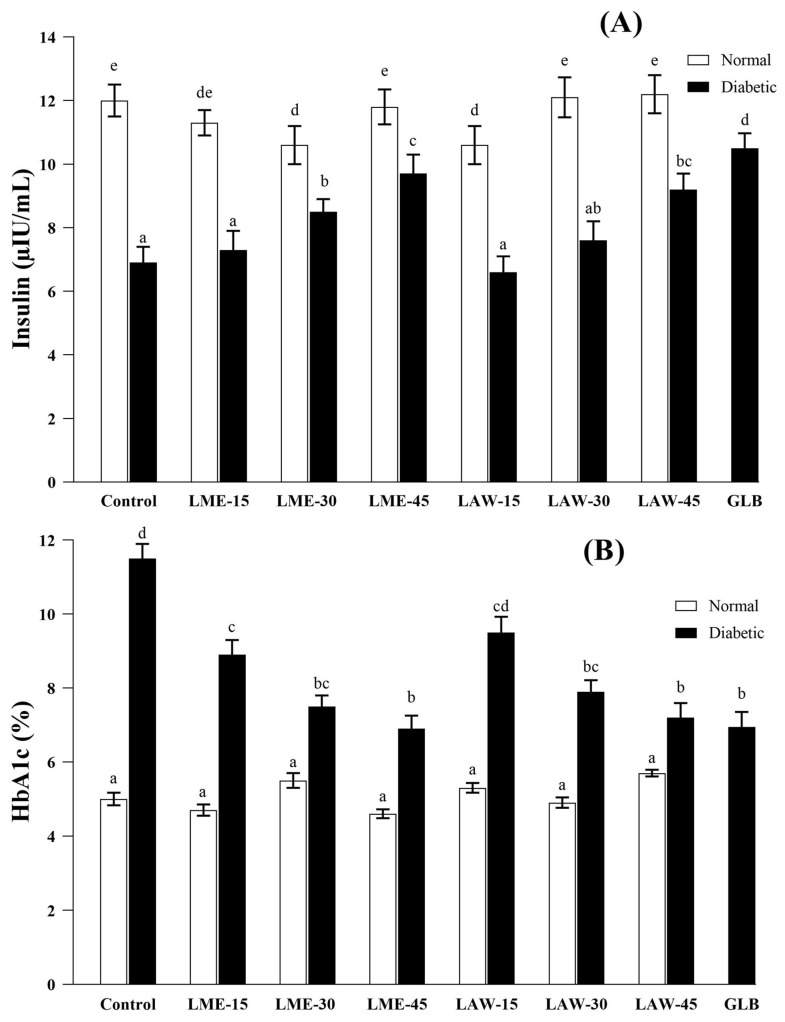
Effects of LME, lawsone (LAW), and glibenclamide (GLB) on insulin (**A**) and HbA1c (**B**) levels. White bars: Normal rats and black-colored bars: Diabetic rats. LME–15, LME–30, and LME–45: 15, 30, and 45 mg/kg of LME, respectively; LAW–15, LAW–30, and LAW–45: 15, 30, and 45 mg/kg of lawsone, respectively; and GLB: 0.6 mg/kg of Glibenclamide. Data are expressed as mean ± S.E.M. (*n* = 6). Based on Duncan’s multiple range test, the values with different letters of the alphabet or superscripts (a–e) indicate significant differences from one another at a significance level of *p* < 0.05. However, the values labeled with the same letters of the alphabet or superscripts indicate no significant differences.

**Figure 3:**
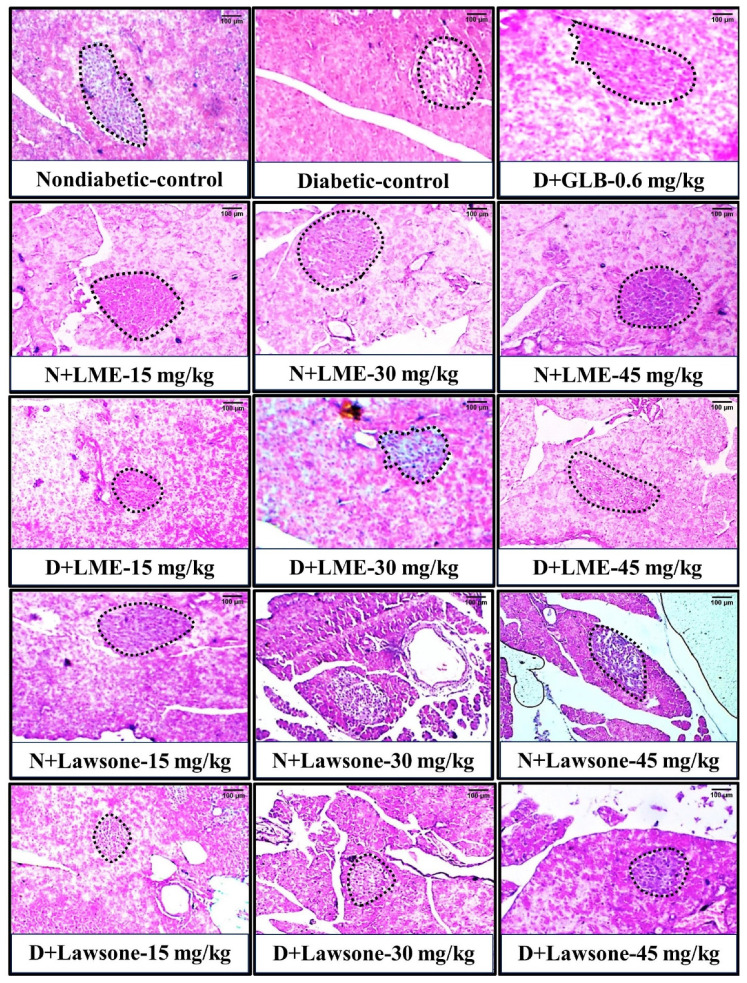
Effects of LME, lawsone, and glibenclamide (GLB) on the apparent size of the islets of Langerhans. Black dashes outline the islets of Langerhans. N + LME or lawsone: Normal rats receiving lawsone methyl ether or lawsone; D + LME or lawsone: Diabetic rats receiving lawsone methyl ether or lawsone; and D + GLB: Diabetic rats receiving glibenclamide. Scale bar = 100 µm.

Figure 1 and Figure 2A indicated that nondiabetic rats receiving cosolvent, LME, and lawsone presented normal FBG and insulin levels over the course of the 28 days. However, diabetic rats treated with 15, 30, and 45 mg/kg of LME and lawsone showed a significant drop in FBG and a marked rise in insulin levels in a dose-dependent manner (Figure 1 and Figure 2A). Glibenclamide (0.6 mg/kg) exhibited the most potent hypoglycemic effect, which was almost identical to the effect generated by LME at its maximal dose (45 mg/kg). However, lawsone’s therapeutic impact on FBG at 45 mg/kg was markedly weaker when compared to the effect of LME at a similar dosage. In fact, lawsone at 45 mg/kg was able to create a hypoglycemic effect that was statistically comparable to LME at 30 mg/kg. This discrepancy in the hypoglycemic potentials of two naphthoquinones is consistent with our previous in vitro study, which found that LME exhibited a higher inhibitory activity on α-glucosidase than lawsone. The hypoglycemic effects of LME and lawsone were correlated with the histopathological images of the pancreas, as depicted in Figure 3. These images apparently illustrated that the islets of Langerhans in the diabetic control group were noticeably shrunk with marked loss of its cells as compared to the normal control group. However, the repeated oral administration of LME and lawsone at doses of 15, 30, and 45 mg/kg resulted in a gradual increase in the size of the islets in the diabetic rats. In addition, both LME and lawsone exhibit pancreatic protective effects, as they have previously been reported to possess promising antioxidant and anti-inflammatory characteristics [35]. These properties suggest that LME and lawsone can mitigate oxidative stress and inflammation within the pancreas, potentially contributing to the preservation and improved function of pancreatic tissues. Moreover, significant hypoglycemic actions have been documented for several other 1,4-naphthoquinones, such as plumbagin, shikonin, and rhinacanthin-C. These compounds, like LME and lawsone, exhibit notable antihyperglycemic effects. The shared hypoglycemic actions among these 1,4-naphthoquinones highlight their potential as a class of compounds for the management of hyperglycemia and related metabolic disorders [15,16,17].

HbA1c is a clinical marker that indicates persistent hyperglycemia. It is widely regarded as the most reliable index for evaluating the long-term effectiveness of antidiabetic medications. HbA1c provides valuable insights into overall glycemic control and serves as an important tool for assessing the long-term efficacy of these drugs in managing diabetes [36]. As shown in Figure 2B, LME and lawsone substantially and dose-dependently decreased the elevated levels of HbA1c in diabetic rats. The most prominent reduction in HbA1c level was observed with 45 mg/kg of LME (6.9 ± 0.5%), which was equivalent to the standard drug, glibenclamide (6.95 ± 0.6%), but likely higher than lawsone (7.2 ± 0.5%) at its maximal dose. In contrast, both LME and lawsone exhibited no effect on the HbA1c levels of normal rats. These results are in line with those of previous studies, which have revealed that different plant-derived phenolics may exert their hypoglycemic effects via antioxidant and anti-glycation mechanisms [37].

The remarkable hypoglycemic effects induced by LME or lawsone are justified through multiple mechanisms. These mechanisms may include enhanced insulin secretion or synthesis, regeneration of pancreatic β-cells, improved insulin sensitivity, and potential modulation of glucose metabolism pathways. In the present study, both LME and lawsone are found to act as insulin secretagogues. This mechanism is supported by a substantial rise in the insulin levels of diabetic rats treated with LME or lawsone (Figure 2A). Both of these compounds may exhibit a mechanism of action similar to glibenclamide by blocking ATP-sensitive potassium channels. This action results in the depolarization of pancreatic β-cells, subsequently leading to the release of insulin [38].

Another mechanism that supports the antihyperglycemic effects of LME and lawsone is their potential to promote the regeneration of pancreatic β-cells. This regenerative action contributes to increased insulin production and secretion. Many antidiabetic drugs including GLP-1 receptor agonists and DPP-4 inhibitors are incretin-based therapies that have shown the potential to enhance β-cell function by promoting their proliferation [39]. The histopathological images of the pancreas from the current study provide apparent support for the aforementioned phenomenon (Figure 3). Observations revealed that the Islets of Langerhans in the diabetic control group were noticeably shrunk with marked loss of its cells as compared to the normal control group. However, the repeated oral administration of LME and lawsone at doses of 15, 30, and 45 mg/kg resulted in a gradual increase in the size of the islets in the diabetic rats. Furthermore, no significant effect was observed on the Islets of Langerhans in normal rats treated with different dosages of LME and lawsone. These findings suggest that LME and lawsone exhibit a potential role in promoting the regeneration or preservation of pancreatic β-cells in diabetic conditions. Nonetheless, it is important to note that β-cell regeneration is a complex process, and more research is needed to fully understand the antidiabetic mechanisms of LME and lawsone in terms of β-cell proliferation.

Additionally, our prior in vitro study showed that LME and lawsone have strong α-glucosidase inhibitory effects. Their hypoglycemic effects can be explained, in part, by the fact that they slow the digestion and absorption of dietary carbohydrates in the gut. Slowing the process of gluconeogenesis or enhancing the uptake and utilization of glucose in adipose tissues, skeletal muscles, and the liver are proposed mechanisms of action for LME and lawsone that warrant further investigation [40]. Additionally, activating peroxisome proliferator-activated receptor gamma (PPARγ) and inhibiting DPP-4 or SGLT-2 can also be the potential antidiabetic mechanisms of LME and lawsone that require further exploration [41]. These mechanisms, if confirmed, could contribute to the overall understanding of the multifaceted therapeutic potentials of LME and lawsone in the management of DM.

### 3.5. Effects of LME and Lawsone on HOMA-IR and HOMA-β

In type 2 DM, two primary characteristics are observed: insulin resistance in peripheral tissues and inadequate insulin production from pancreatic β-cells. These factors, insulin resistance and insulin secretion, are commonly quantified using HOMA-IR and HOMA-β, respectively. Both HOMA indicators are clinically related to one another and have been carefully linked to the etiology, progression, and complications of DM [42]. HFFD- and NA-STZ-induced diabetic control rats exhibited a fivefold increase in insulin resistance (HOMA-IR) and a ninefold decrease in β-cell function (HOMA-β) as compared to the normal control rats (Table 3). The oral administration of LME and lawsone for 28 days showed a dose-dependent decrease in HOMA-IR and a dose-dependent increase in HOMA-β. However, there was no significant alteration in the HOMA indices for the normal rats treated with the same doses of LME and lawsone. The significant normalization of HOMA indicators (HOMA-IR and HOMA-β) observed after the administration of LME and lawsone suggests an improvement in glucose homeostasis. This normalization indicates an enhancement in insulin action, leading to increased insulin sensitivity, as well as an improvement in β-cell function. These findings imply that LME and lawsone may have the potential to improve the overall regulation of glucose levels by enhancing both insulin action and β-cell function.

### 3.6. Effects of LME and Lawsone on Lipid Profiles

DM has an intimate link with hyperlipidemia. This often leads to cardiomyopathy, a major cause of mortality among diabetics [43]. In the present study, a similar positive correlation was observed between hyperglycemia and hyperlipidemia. An ideal animal model of diabetic hyperlipidemia should closely resemble the human condition. One approach to achieve this is by inducing obesity/hyperlipidemia through dietary intervention and causing pancreatic damage via nicotinamide-streptozotocin. This combination better reflects the human scenario and provides a more suitable representation of diabetic hyperlipidemia in rats. Similarly, in the present study, an ideal model of diabetic hyperlipidemia was created in rats using NA-STZ injection in conjunction with a high-fat (45% fat by energy) and high-fructose (30% fructose in drinking water ad libitum) diet. Insulin deficiency is primarily responsible for diabetic hyperlipidemia. It instigates hormone-sensitive lipase that promotes lipolysis and boosts the release of free fatty acids [44]. Excessive fatty acids enhance the production of TC, TG, and LDL while decreasing HDL biosynthesis, as observed in untreated diabetic rats of the current study.

Regular oral administration of LME and lawsone (15, 30, and 45 mg/kg) for 28 days delivered a substantial and dose-dependent amelioration of deteriorated effects on TC, TG, LDL, and HDL (Table 4). However, normal rats, which were given similar doses of LME and lawsone, showed no discernible change in their lipid profiles. LME at 45 mg/kg seemed to be the most effective in normalizing TC, HDL, and LDL levels in diabetic rats, which were better than the standard drug, glibenclamide. These findings suggest that LME has potent therapeutic benefits in improving lipid profiles in diabetic conditions, surpassing the efficacy of glibenclamide in this particular study. Lawsone at 45 mg/kg was the most potent in lowering the TG level without any distinguishable impact on other lipid parameters. The hypolipidemic effects of LME and lawsone are attributed to their potential for stimulation of residual β-cells. This stimulation could result in increased insulin synthesis, secretion, or sensitivity. The precise mechanism underlying these effects requires further investigation. However, it is hypothesized that the improvement in lipid profiles seen with LME and lawsone could be related to their impact on β-cell function and insulin-related pathways, which ultimately contribute to the regulation of lipid metabolism in the body. The findings of this study align with previous research that has demonstrated the hypolipidemic properties of other 1,4-naphthoquinones, including plumbagin, shikonin, and rhinacanthin-C [15,16,17].

Dyslipidemia is associated with an increased risk of cardiovascular pathology, which is quantified by measuring atherogenicity indicators, such as the AI, AC, and CRI. As illustrated in Table 5, the diabetic control rats had noticeably higher atherogenicity risk indicators compared to the normal control rats. A substantial and dose-dependent reduction in atherogenicity indicators was observed in diabetic rats treated with LME/lawsone (15, 30, and 45 mg/kg) for 28 days. LME at 45 mg/kg caused the most potent decline in atherogenic indicators, followed by glibenclamide (0.6 mg/kg) and lawsone (45 mg/kg). The most striking impact of LME (45 mg/kg) was found on CRI, decreasing it to a level of 2.1 ± 0.2, which was significantly comparable with that of the normal level (1.78 ± 0.2). Indeed, numerous studies have reported that antioxidant and anti-inflammatory agents derived from plants have the ability to protect against the formation and progression of atherosclerosis, which is the underlying cause of atherogenicity [45]. Thus, lowering of the aforesaid markers is an indication of the cardioprotective potential of LME and lawsone due to their antioxidant, anti-inflammatory, and antihyperlipidemic potentials. By targeting these mechanisms, LME and lawsone demonstrate potential as therapeutic agents for promoting heart health and reducing the risk of cardiovascular complications in diabetic patients.

### 3.7. Effects of LME and Lawsone on Liver and Kidney Function Parameters

The liver maintains healthy blood glucose levels by performing various metabolic processes, including glycogenesis, glycogenolysis, and gluconeogenesis. AST and ALT are the enzymes, which are the indicative markers of liver health. Due to a liver injury, they may seep into the bloodstream, leading to their increased levels [46]. In the current study, increases in levels of AST and ALT were observed in the untreated diabetic rats, indicating an altered liver function that can be linked to hyperglycemia. The higher levels of AST and ALT in diabetic rats were significantly reduced by LME/lawsone (15, 30, and 45 mg/kg) in a dose-dependent manner (Table 6). Amongst these, lawsone at 45 mg/kg caused the maximum reduction in AST and ALT levels in diabetic livers, followed by LME (45 mg/kg) and glibenclamide (0.6 mg/kg). On the other hand, LME and lawsone did not significantly alter the liver biomarkers in normal rats. In the present study, lawsone demonstrated the most significant reduction in liver functional markers. These findings align with previous reports highlighting the hepatoprotective effects of lawsone [47]. Moreover, the prior studies on lawsone and LME have provided additional evidence supporting their hepatoprotective effects, specifically due to their promising antioxidant and anti-inflammatory activities [35].

Long-term uncontrolled hyperglycemia may result in diabetic nephropathy, which is one of the serious complications of DM. It develops from undue oxidative stress that is induced by an excess of free radicals [48]. In nephropathic conditions, serum creatinine and BUN levels are useful markers to assess the functional efficiency of kidneys [49]. In this study, elevated levels of creatinine and BUN were observed in untreated diabetic rats, indicating impaired renal function resulting from hyperglycemia. The elevated levels of creatinine and BUN in diabetic rats were significantly reduced by LME/lawsone (15, 30, and 45 mg/kg) in a dose-dependent manner (Table 6). The highest decline in the renal biomarkers of diabetic rats was observed with LME at a dose of 45 mg/kg, followed by glibenclamide at 0.6 mg/kg and lawsone at 45 mg/kg. Neither LME nor lawsone significantly altered the renal biomarkers in normal rats. These findings are consistent with the antioxidant and anti-inflammatory properties of LME and lawsone [34]. These results suggest that LME and lawsone not only demonstrate efficacy as hypoglycemic agents but also have the potential to ameliorate diabetic nephropathy. These effects of LME and lawsone are similar to those observed with another 1,4-naphthoquinone compound, rhinacanthin-C [50]. The ability of LME, lawsone, and rhinacanthin-C to exhibit hypoglycemic and nephroprotective properties further highlights the potential of 1,4-naphthoquinones in managing diabetes and its related complications.

## 4. Conclusions

The present study supports that LME is a potent antidiabetic drug. Furthermore, the study suggests that LME is also a potential compound to mitigate the lethal complications associated with DM due to its promising antihyperlipidemic, hepatoprotective, and nephroprotective properties. The optimum dose of LME possessing maximum efficacy and safety was found to be 45 mg/kg. Interestingly, LME was found to be more potent and safer than lawsone. Various mechanisms could define the antidiabetic potential of LME; however, in the present study, LME was found to act via α-glucosidase inhibition and pancreatic β-cell regeneration. These findings highlight the multifaceted therapeutic potential of LME in managing DM along with its complications, thereby emphasizing its significance as a potential treatment option. Nevertheless, further research is warranted to explore the antidiabetic mechanisms of LME using various pathways and targets involved in glucose regulation, such as gluconeogenesis, glucose transportation, ATP-sensitive potassium channels, incretin hormones, and ghrelin.

## Figures and Tables

**Table 1:** Body weight changes and serum biochemical indices of rats fed on NFD and HFFD.

Parameters	Before Diet Intervention		After 10 Weeks of Diet Intervention	
	NFD	HFFD	NFD	HFFD
Weight (g)	132.8 ± 3.9 ^a^	125.7 ± 4.3 ^a^	193.9 ± 6.2 ^b^	214.1 ± 6.0 ^c^
BWG (g)	N/A	N/A	61.7 ± 6.0	88.6 ± 6.9
TC (mg/dL)	78.6 ± 5.1 ^a^	84.3 ± 5.9 ^a^	89.1 ± 4.3 ^a^	113.3 ± 7.8 ^b^
TG (mg/dL)	57.9 ± 3.7 ^a^	60.6 ± 5.5 ^a^	68.4 ± 6.0 ^a^	97.1 ± 6.2 ^b^
HDL (mg/dL)	28.3 ± 2.6 ^ab^	32.9 ± 3.0 ^b^	34.8 ± 3.3 ^b^	21.4 ± 1.9 ^a^
LDL (mg/dL)	8.5 ± 1.0 ^a^	11.9 ± 0.9 ^a^	12.3 ± 1.3 ^a^	18.9 ± 2.1 ^b^
FBG (mg/dL)	82.8 ± 4.6 ^a^	89.6 ± 4.1 ^ab^	91.4 ± 5.1 ^ab^	103 ± 5.6 ^b^
Insulin (µIU/mL)	9.9 ± 0.7 ^a^	11.3 ± 0.4 ^a^	12.0 ± 0.8 ^a^	10.8 ± 1.2 ^a^

NFD: Normal fat diet, HFFD: High-fat/high-fructose diet, BWG: Body weight gain, N/A: Not available. Data are expressed as mean ± S.E.M. (*n* = 6). Based on Duncan’s multiple range test, the values with different letters of the alphabet or superscripts (a–c) indicate significant differences from one another at a significance level of *p* < 0.05. However, the values labeled with the same letters of the alphabet or superscripts indicate no significant differences.

**Table 2:** Effects of LME and lawsone on body weight, food, and water intake in rats.

Group	Compound Dose	Parameters				
		Initial BW (g)	Final BW (g)	BWG (g)	Food/day (g)	H_2_O/day (mL)
Normal	Control	197.6 ± 5.1 ^b^	233.7 ± 7.7 ^bc^	36.1	31.1 ± 3.1 ^ab^	22.1 ± 1.9 ^a^
	LME—15 mg/kg	189.4 ± 4.6 ^ab^	216.9 ± 7.5 ^ab^	27.5	28.6 ± 2.7 ^a^	19.9 ± 1.5 ^a^
	LME—30 mg/kg	189.5 ± 5.0 ^ab^	221.3 ± 6.6 ^b^	31.8	31.9 ± 3.4 ^ab^	21.2 ± 1.4 ^a^
	LME—45 mg/kg	199.6 ± 5.6 ^b^	238.4 ± 8.1 ^bc^	38.8	38.1 ± 4.2 ^b^	24.2 ± 1.8 ^ab^
	Lawsone—15 mg/kg	198.8 ± 4.9 ^b^	225.1 ± 6.7 ^b^	26.2	30.1 ± 2.9 ^ab^	20.3 ± 1.6 ^a^
	Lawsone—30 mg/kg	178.5 ± 3.6 ^a^	211.7 ± 6.1 ^ab^	33.2	33.4 ± 3.3 ^ab^	23.1 ± 2.1 ^ab^
	Lawsone—45 mg/kg	203.9 ± 6.1 ^abc^	241.3 ± 8.7 ^bc^	37.4	37 ± 3.9 ^b^	24.9 ± 2.0 ^ab^
Diabetic	Control	218.9 ± 6.3 ^c^	194.1 ± 6.1 ^a^	−24.8	64.9 ± 4.8 ^d^	40.9 ± 3.7 ^c^
	LME—15 mg/kg	213.3 ± 7.0 ^bc^	227.9 ± 7.3 ^b^	14.6	50.1 ± 4.5 ^cd^	35.3 ± 2.9 ^bc^
	LME—30 mg/kg	208.4 ± 4.5 ^bc^	234.5 ± 7.9 ^bc^	26.1	45.3 ± 3.9 ^c^	30.8 ± 3.3 ^abc^
	LME—45 mg/kg	221.1 ± 7.1 ^c^	257.8 ± 8.9 ^c^	36.7	40.8 ± 3.4 ^bc^	29.9 ± 2.7 ^abc^
	Lawsone—15 mg/kg	209.4 ± 5.1 ^bc^	222.3 ± 4.9 ^b^	12.9	53.7 ± 4.2 ^cd^	39.1 ± 3.5 ^c^
	Lawsone—30 mg/kg	217.1 ± 6.5 ^bc^	239.2 ± 8.1 ^bc^	22.1	46.9 ± 4.5 ^bc^	38.2 ± 2.9 ^c^
	Lawsone—45 mg/kg	205.7 ± 5.6 ^bc^	238.1 ± 7.3 ^bc^	32.4	41.3 ± 3.0 ^bc^	30.8 ± 3.1 ^abc^
	Glb—0.6 mg/kg	219.2 ± 6.9 ^c^	257.1 ± 9.1 ^c^	37.9	39.9 ± 3.6 ^bc^	29.7 ± 2.8 ^abc^

LME: Lawsone methyl ether, BW: Body weight, BWG: Body weight gain, Glb: Glibenclamide. Data are expressed as mean ± S.E.M. (*n* = 6). Based on Duncan’s multiple range test, the values with different letters of the alphabet or superscripts (a–d) indicate significant differences from one another at a significance level of *p* < 0.05. However, the values labeled with the same letters of the alphabet or superscripts indicate no significant differences.

**Table 3:** Effects of LME and lawsone on HOMA indices of adult male Wistar rats.

Group	Compound Dose	Parameters	
		HOMA Insulin Resistance	HOMA β-Cells Function
Normal	Control	2.7 ± 0.1 ^a^	126.1 ± 7.5 ^f^
	LME—15 mg/kg	2.2 ± 0.2 ^a^	172.3 ± 5.6 ^h^
	LME—30 mg/kg	2.1 ± 0.2 ^a^	164.9 ± 4.8 ^g^
	LME—45 mg/kg	2.5 ± 0.1 ^a^	137.3 ± 4.6 ^f^
	Lawsone—15 mg/kg	2.4 ± 0.3 ^a^	125.4 ± 7.9 ^f^
	Lawsone—30 mg/kg	2.5 ± 0.1 ^a^	144.4 ± 8.6 ^g^
	Lawsone—45 mg/kg	2.4 ± 0.2 ^a^	184.6 ± 6.9 ^h^
Diabetic	Control	9.9 ± 0.5 ^d^	13.9 ± 0.9 ^a^
	LME—15 mg/kg	4.5 ± 0.6 ^c^	21.1 ± 1.0 ^ab^
	LME—30 mg/kg	3.5 ± 0.3 ^bc^	40.8 ± 5.3 ^c^
	LME—45 mg/kg	2.9 ± 0.3 ^b^	67.6 ± 4.1 ^e^
	Lawsone—15 mg/kg	4.9 ± 0.6 ^c^	17.5 ± 1.4 ^a^
	Lawsone—30 mg/kg	3.8 ± 0.5 ^bc^	31.9 ± 2.9 ^b^
	Lawsone—45 mg/kg	3.2 ± 0.1 ^bc^	51.5 ± 3.4 ^cd^
	Glb—0.6 mg/kg	3.0 ± 0.4 ^b^	69. 4 ± 3.9 ^e^

LME: Lawsone methyl ether, Glb: Glibenclamide. Data are expressed as mean ± S.E.M. (*n* = 6). Based on Duncan’s multiple range test, the values with different letters of the alphabet or superscripts (a–h) indicate significant differences from one another at a significance level of *p* < 0.05. However, the values labeled with the same letters of the alphabet or superscripts indicate no significant differences.

**Table 4:** Effects of LME and lawsone on lipid profiles in adult male Wistar rats.

Group	Compound Dose	Parameters (mg/dL)			
		TC	TG	HDL	LDL
Normal	Control	92.6 ± 5.2 ^ab^	76.2 ± 6.1 ^ab^	50.8 ± 6.1 ^bc^	11.2 ± 1.2 ^a^
	LME—15 mg/kg	94.8 ± 5.9 ^ab^	68.6 ± 5.0 ^a^	48.6 ± 5.0 ^bc^	10.6 ± 1.4 ^a^
	LME—30 mg/kg	97.8 ± 6.9 ^ab^	71.6 ± 5.4 ^a^	50.6 ± 4.8 ^bc^	10.7 ± 0.8 ^a^
	LME—45 mg/kg	88.9 ± 4.3 ^a^	78.2 ± 4.9 ^ab^	49.8 ± 5.1 ^bc^	11.9 ± 1.1 ^a^
	Lawsone—15 mg/kg	94.9 ± 4.8 ^ab^	74.8 ± 4.2 ^ab^	47.9 ± 4.0 ^bc^	11.5 ± 1.4 ^a^
	Lawsone—30 mg/kg	101.2 ± 6.8 ^ab^	81.2 ± 5.1 ^ab^	54.7 ± 3.9 ^c^	12.1 ± 0.9 ^a^
	Lawsone—45 mg/kg	89.0 ± 6.5 ^a^	80 ± 6.9 ^ab^	45.9 ± 3.8 ^abc^	10.3 ± 1.2 ^a^
Diabetic	Control	201 ± 11.1 ^f^	168.4 ± 8.2 ^e^	28.6 ± 4.7 ^a^	71.8 ± 5.9 ^g^
	LME—15 mg/kg	167.8 ± 9.5 ^de^	147.2 ± 7.4 ^e^	35.1 ± 4.2 ^ab^	50.8 ± 5.6 ^ef^
	LME—30 mg/kg	122.6 ± 7.9 ^bc^	113.2 ± 5.4 ^cd^	42.2 ± 3.8 ^abc^	31.6 ± 3.4 ^cd^
	LME—45 mg/kg	109 ± 5.7 ^ab^	95.4 ± 4.9 ^bc^	50.5 ± 5.7 ^bc^	16.5 ± 2.2 ^ab^
	Lawsone—15 mg/kg	184.4 ± 8.9 ^ef^	145.9 ± 6.9 ^e^	34.8 ± 3.9 ^ab^	57.8 ± 4.4 ^f^
	Lawsone—30 mg/kg	151.8 ± 7.1 ^cd^	119.4 ± 6.3 ^d^	40.8 ± 5.0 ^abc^	38.9 ± 4.3 ^de^
	Lawsone—45 mg/kg	121.2 ± 6.4 ^b^	89.4 ± 4.9 ^ab^	45.4 ± 4.7 ^abc^	27.6 ± 2.2 ^bcd^
	Glb—0.6 mg/kg	116.2 ± 5.1 ^ab^	96. 2 ± 5.4 ^bc^	48.6 ± 5.1 ^bc^	19.8 ± 1.6 ^abc^

LME: Lawsone methyl ether, Glb: Glibenclamide. Data are expressed as mean ± S.E.M. (*n* = 6). Based on Duncan’s multiple range test, the values with different letters of the alphabet or superscripts (a–f) indicate significant differences from one another at a significance level of *p* < 0.05. However, the values labeled with the same letters of the alphabet or superscripts indicate no significant differences.

**Table 5:** Effects of LME and lawsone on atherogenicity indicators for adult male Wistar rats.

Group	Compound Dose	Atherogenicity Indicators		
		AI	AC	CRI
Normal	Control	0.82 ± 0.1 ^a^	0.22 ± 0.02 ^a^	1.82 ± 0.2 ^a^
	LME—15 mg/kg	0.95 ± 0.1 ^a^	0.21 ± 0.03 ^a^	1.95 ± 0.1 ^a^
	LME—30 mg/kg	0.93 ± 0.2 ^a^	0.2 ± 0.01 ^a^	1.93 ± 0.2 ^a^
	LME—45 mg/kg	0.81 ± 0.1 ^a^	0.23 ± 0.02 ^a^	1.78 ± 0.2 ^a^
	LAW—15 mg/kg	0.98 ± 0.2 ^a^	0.24 ± 0.03 ^a^	1.98 ± 0.3 ^a^
	LAW—30 mg/kg	0.85 ± 0.09 ^a^	0.18 ± 0.01 ^a^	1.85 ± 0.2 ^a^
	LAW—45 mg/kg	0.93 ± 0.1 ^a^	0.22 ± 0.03 ^a^	1.93 ± 0.1 ^a^
Diabetic	Control	6.1 ± 0.9 ^e^	2.51 ± 0.3 ^e^	7.02 ± 1.0 ^d^
	LME—15 mg/kg	3.8 ± 0.4 ^cd^	1.44 ± 0.1 ^d^	4.78 ± 0.3 ^c^
	LME—30 mg/kg	1.9 ± 0.2 ^ab^	0.74 ± 0.05 ^bc^	2.9 ± 0.3 ^ab^
	LME—45 mg/kg	1.15 ± 0.1 ^ab^	0.32 ± 0.04 ^ab^	2.1 ± 0.2 ^a^
	LAW—15 mg/kg	4.3 ± 0.5 ^d^	1.66 ± 0.2 ^d^	5.29 ± 0.5 ^c^
	LAW—30 mg/kg	2.7 ± 0.3 ^bc^	0.95 ± 0.1 ^c^	3.72 ± 0.4 ^bc^
	LAW—45 mg/kg	1.66 ± 0.2 ^ab^	0.6 ± 0.07 ^abc^	2.66 ± 0.2 ^ab^
	GLB—0.6 mg/kg	1.39 ± 0.2 ^ab^	0.4 ± 0.06 ^ab^	2.39 ± 0.3 ^ab^

LME: Lawsone methyl ether, Glb: Glibenclamide. Data are expressed as mean ± S.E.M. (*n* = 6). Based on Duncan’s multiple range test, the values with different letters of the alphabet or superscripts (a–e) indicate significant differences from one another at a significance level of *p* < 0.05. However, the values labeled with the same letters of the alphabet or superscripts indicate no significant differences.

**Table 6:** Effects of LME and lawsone on liver and kidney functions in adult male Wistar rats.

Group	Compound Dose	Parameters (mg/dL)			
		AST (IU/dL)	ALT (IU/dL)	Cr (mg/dL)	BUN (mg/dL)
Normal	Control	88.6 ± 6.0 ^a^	44.0 ± 3.6 ^ab^	0.39 ± 0.06 ^a^	28.1 ± 3.2 ^a^
	LME—15 mg/kg	90.9 ± 5.7 ^ab^	39.8 ± 3.0 ^a^	0.38 ± 0.04 ^a^	26.9 ± 3.0 ^a^
	LME—30 mg/kg	88.4 ± 6.6 ^a^	41.8 ± 5.1 ^ab^	0.40 ± 0.06 ^a^	30.9 ± 3.7 ^abc^
	LME—45 mg/kg	92.3 ± 5.4 ^ab^	38.6 ± 4.1 ^a^	0.41 ± 0.05 ^a^	31.6 ± 4.3 ^abc^
	Lawsone—15 mg/kg	89.3 ± 6.1 ^a^	47.9 ± 4.7 ^ab^	0.45 ± 0.06 ^ab^	28.6 ± 3.2 ^ab^
	Lawsone—30 mg/kg	99.0 ± 7.6 ^abc^	51.1 ± 4.3 ^ab^	0.37 ± 0.03 ^a^	33.8 ± 2.9 ^abc^
	Lawsone—45 mg/kg	100.8 ± 7.3 ^abc^	40.3 ± 63.5 ^a^	0.40 ± 0.05 ^ab^	30.8 ± 4.0 ^abc^
Diabetic	Control	169.8 ± 11.1 ^f^	98.4 ± 6.0 ^g^	1.1 ± 0.09 ^e^	61.9 ± 5.1 ^e^
	LME—15 mg/kg	157.8 ± 7.3 ^ef^	89.1 ± 5.4 ^fg^	0.85 ± 0.06 ^cd^	53.4 ± 4.3 ^de^
	LME—30 mg/kg	136.6 ± 5.3 ^de^	75.6 ± 6.0 ^cdef^	0.61 ± 0.03 ^abc^	45.6 ± 4.0 ^cde^
	LME—45 mg/kg	118.9 ± 5.7 ^bcd^	60.1 ± 3.6 ^abcd^	0.39 ± 0.04 ^a^	33.8 ± 3.0 ^abc^
	Lawsone—15 mg/kg	153.9 ± 7.6 ^ef^	85.3 ± 4.6 ^efg^	0.86 ± 0.09 ^d^	54.9 ± 5.1 ^de^
	Lawsone—30 mg/kg	139.6 ± 5.3 ^de^	79.9 ± 5.4 ^defg^	0.73 ± 0.07 ^bcd^	45.0 ± 4.3 ^bcd^
	Lawsone—45 mg/kg	117.8 ± 7.5 ^bcd^	54.2 ± 4.5 ^abc^	0.51 ± 0.05 ^ab^	41.9 ± 3.7 ^abcd^
	Glb—0.6 mg/kg	120.2 ± 6.5 ^cd^	63. 6 ± 5.5 ^bcde^	0.42 ± 0.04 ^a^	35.1 ± 4.1 ^abc^

LME: Lawsone methyl ether, Cr: Creatinine, Glb: Glibenclamide. Data are expressed as mean ± S.E.M. (*n* = 6). Based on Duncan’s multiple range test, the values with different letters of the alphabet or superscripts (a–g) indicate significant differences from one another at a significance level of *p* < 0.05. However, the values labeled with the same letters of the alphabet or superscripts indicate no significant differences.

## Data Availability

The data presented in this study are available in article.
